# A Multidisciplinary Approach in Prenatal Diagnosis of TSC With Cardiac Rhabdomyoma as the Initial Symptom

**DOI:** 10.3389/fped.2021.628238

**Published:** 2021-08-27

**Authors:** Yiming Qi, Hongke Ding, Yanlin Huang, Yukun Zeng, Lihua Yu, Ling Liu, Yan Zhang, Aihua Yin

**Affiliations:** ^1^Prenatal Diagnosis Centre, Guangdong Women and Children Hospital, Guangzhou, China; ^2^Maternal and Children Metabolic-Genetic Key Laboratory, Guangdong Women and Children Hospital, Guangzhou, China

**Keywords:** cardiac rhabdomyoma, prenatal diagnoses, tuberous sclerosis (TSC), fetal tumor, targeted exome capture sequencing

## Abstract

The long-term prognosis of a fetus with cardiac rhabdomyoma (CR) depends on the correlation with tuberous sclerosis complex (TSC). In recent years, the numerous variations of uncertain significance (VUS) of TSC genes produced by high-throughput sequencing have made counseling challenging, studies until now have tended to side-step the tricky topics. Here, we integrated detailed parental phenotype, echocardiography, neuro MRI, and genetic information to conduct a comprehensive evaluation of 61 CR fetuses. As a result, multiple CRs and cerebral lesions appeared in 90 and 80%, respectively of fetuses with pathogenic (P)/likely pathogenic (LP) TSC1/TSC2 variations. Overall, 85.7% of the live-born infants with P/LP presented with TSC-associated signs. While, 85.7% of VUS without nervous findings had good prognoses. Genetic evidence and cerebral MRI findings are the most sensitive index to assess long-term prognosis, which complement and confirm each other for a TSC diagnosis. In total, 68.9% of fetuses with CR could benefit from this multidisciplinary approach, which turned out to be potentially clinically actionable with precise clinical/genetic diagnosis or had a foreseeable outcome. Our practice provides a practical and feasible solution for perinatal management and prognostic guidance for fetuses with CR.

## Introduction

Cardiac rhabdomyoma (CR) is one of the most common primary cardiac tumors which occurs predominately in infants and children. The reported incidence varies from 0.02 to 0.17% in live-born infants, and 0.12% in prenatal fetal studies (1, 2). An unprecedented increase in CR has benefited from the rapid development of fetal echocardiography in recent decades. However, it is still challenging to obtain accurate prognostic information of these newborns.

As a benign hamartoma, the prognosis of CR without severe complications is typically good (3). But CR is unpredictable and occurs anywhere in the heart, which may result in arrhythmias, effusions, fetal hydrops, and even stillbirth. It is worth noting, to some extent, that CR is a characteristic sign and an initial symptom for tuberous sclerosis complex (TSC) prenatally, which has a high possibility of neurological development disorder. Reported rates of TSC in fetuses with CR vary from 50 to 90% (4, 5). Approximately 90% of infants with TSC experience infantile spasms, seizures, intellectual disability, or autism (6). As a multisystem genetically based neurocutaneous syndrome, TSC is inherited in an autosomal dominant manner. Cortical tubers and subependymal nodules (SEN) are typical cerebral lesions with a high risk of obstructive hydrocephalus, which are the leading cause of mortality. There are few reports about the intrauterine brain damages of TSC, and the relationship with imaging phenotype and genotype is not clear yet.

*TSC1/TSC2* mutations are the vital disease-causing genes and are recognized as independent and sufficient diagnostic criteria, even in the condition of no visible typical clinical signs (7). With the application of NGS technology, the number of identified variants has increased explosively, meanwhile massive VUS have not been far behind. Overall, 3446 *TSC1* and 8112 *TSC2* variations have been recorded in the LOVD Database (http://chromium.lovd.nl/LOVD2/TSC/home), including 268 VUS of *TSC1* and 642 VUS of *TSC2*. They show a broad spectrum of variations without conspicuous hotspots in patients. Mutation types have almost equal coverage including insertions/deletions/duplications, frameshift, non-sense, missense, splicing, and sometimes encounter deep intron mutations (8). This implies that no typical mutation type was well-tolerated. Disproportionately, limited understanding of the role and lack of awareness of genotype-phenotype correlations excessively disturbs our judgment of the pathogenesis with these VUS, which is hugely critical for fetal prognosis, informed reproductive choices, and evidence-based perinatal management.

The unpredictability of the fetal CR and the uncertainty of variations in *TSC1/TSC2* gene makes made phenotype and prognosis prediction of TSC clinically challenging. Here, we revealed complete imaging and the molecular portrait of a fetus with CR(s) to date, adding multi-characteristic information for a comprehensive evaluation and proposing the clinical workflow for clinical management.

## Materials and Methods

### Patient Recruitment and Sample Collection

The study was approved by the institutional review board of the ethics committee in Guangdong Women and Children Hospital (201601046). Pregnant women with suspected fetal CR by the second-trimester screening were recruited in our center from March 2014 to November 2019. All fetuses underwent electrocardiography to evaluate the properties of tumors, and part of them performed brain magnetic resonance imaging (MRI) scans to gather more clinical evidence. A comprehensive clinical examination was concurrently performed in pedigree. The diagnostic criteria we referred to are the international recommendations of TSC, revised in 2013 (7).

Fetal samples were collected from amniocentesis (16–24 weeks) or cordocentesis (more than 24 weeks). Karyotype and SNP-array were performed simultaneously with NGS or before. All the fetuses accompanied with chromosome aneuploidy or copy number variations (CNVs) were excluded. For each relevant family member, 5 ml peripheral blood samples were draw and stored in ethylenediaminetetraacetic acid (EDTA) anti-coagulation vacuum tubes. Skin or cardiac tissues from aborted fetuses were collected after signed informed consent was obtained from the enrolled families.

### Fetal Echocardiography and Brain MRI Examinations

According to the “ISUOG Practice Guide: Fetal Heart Ultrasound Screening (Updated Version),” we comprehensively used two-dimensional ultrasound and echocardiography by the GE VoIuson E8 color Doppler ultrasound system (GE Medical Systems, Zipf, Austria) which was equipped with a 2–5 MHz two-dimensional convex array transducer and a 4–8.5 MHz three-dimensional transducer. The tumor number, location, size, echo, relationship with surrounding tissues, heart rhythm, and hemodynamic changes in details, as well as fetal brain, spine, chest, abdomen, limbs and other structures were recorded.

Fetal brain MRI was performed on a Sonata scanner (Siemens, Erlangen, Germany) using a body phased-array coil. Breath-hold imaging was performed optionally without sedation in the supine position. Images were acquired using a T2-W HASTE (half-acquisition single-shot turbo-spin-echo) sequence (TR/TE 1,260/74 ms and matrix 256) and a T1-W gradient-echo sequence (TR/TE 130–160 ms/5–10 ms, matrix 128 and α70). Slice thickness was 4 mm in the axial plane.

### Amniocentesis and Cordocentesis

The procedure was performed using an aseptic technique with infiltration of local anesthesia, under ultrasound guidance, using a real-time ultrasound machine equipped with a 2–5-MHz transabdominal probe. For amniocentesis, the ultrasound probe may be tilted up to 45°, with respect to the mid-sagittal plane of the maternal abdomen, away from the intended side of needle entry, whilst remaining in the same transverse plane. Once the needle was located correctly in the uterine cavity, the stylet was removed and the syringe, or a Luer adapter attached to a Vacutainer® holder, was connected. Finally, ~10–20 ml of amniotic fluid was drawn. For cordocentesis, a 22- or 23-gauge spinal needle was used. The puncture site, either near the cord insertion or in a free-floating loop, was chosen according to the accessibility and quality of visualization, regardless of location. Then, about 4 ml of cord blood was collected, ideally without contamination by maternal blood cells.

### Follow-Up

**Figure 1:**
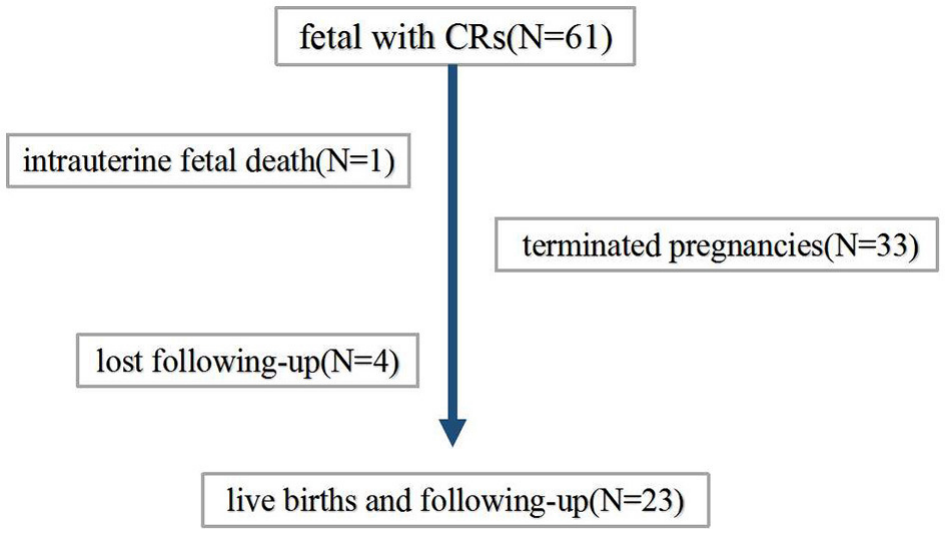
Patient flow diagram.

The patient flow diagram is depicted in Figure 1. Continuing pregnancies were observed, and infants with CRs were born at the author's center or the referral center. All children born were followed up by a pediatric expert team which included a pediatric cardiologist, a pediatric neurologist, an imaging expert, and a genetic counselor. Termination of pregnancy (TOP) or obtaining a diagnosis (clinically or/and genetically) was the endpoint of clinical observation. Postnatal examinations included clinical examination, echocardiography, and MRI scanning if suspicious extra cardiac complications were found.

### Next-Generation Sequencing

Genomic DNA samples were extracted using the Qiagen DNA Blood Midi/Mini kit (Qiagen GmbH, Hilden, Germany) according to the manufacturer's instructions. Sequencing of the *TSC1* gene and *TSC2* gene in Trios was performed with a custom-designed Roche Nimblegen (Madison, WI) (SeqCap EZ Choice Library) chip capturing the *TSC1* (NM_000368, exon 3-23, exons 1 and 2 are UTRs) and *TSC2* (NM_000548, exon 2-42, exons 1 UTRs) genes following the manufacturer's protocols (JIAJIAN Clinical Laboratories, BEIJING, China). The found variants were further verified by Sanger sequencing in fetuses and parents. Analysis of genetic results was based on the genomic variation database (http://dgv.tcag.ca/dgv/app/home), DECIPHER database (https://decipher.sanger.ac.uk/), and OMIM database (http://www.ncbi.nlm.nih.gov/omim). Variant classification was based on the guidelines of the ACMG and divided into five categories: “I: pathogenic (P),” “II: likely pathogenic (LP),” “III: variants of uncertain significance (VUS),” “IV: likely benign (LB),” and “V: benign (B).”

## Results

### General Information and Imaging Results Prenatally

A total of 61 fetuses with CR were included in the study. The median age of pregnant women was 28 (range 22–38) years. The average GA of CRs detected during routine scans was 25 ± 3.5 weeks (range 16–34). There were 59 single pregnancies and two dichorionic twin pregnancies, in which one of the fetuses presented with CR. Sonographic images showed round, hyperechogenic, homogenous masses with clear boundaries and absence of color in the Doppler flow inner. Solitary and multiple rhabdomyomas were detected in 18 fetuses (29.5%) and 43 fetuses (70.5%), respectively (Supplementary Figures 1A,B). All CRs were distributed in the ventricle, interventricular septum, and the atrium; the most common areas were the left ventricle and ventricular septum. Tumor size was recorded in 59 cases, and, of these, the median diameter was 8.1 mm (range 2.2–31.8). Forty-nine fetuses had more than one echocardiography monitoring record. Throughout the pregnancy, in 41% of the 49 fetuses tumors increased, seven cases kept stable, and only one had somehow subsided. Echocardiographic findings are summarized in Table 1.

**Table 1:** Summary of echocardiographic findings in fetuses with CR.

**Number of tumors**	***n* (%)**
Solitary	18 (29.5%)
Multiple	43 (70.5%)
**Location of tumors**	
LV	46 (75.4%)
RV	29 (47.5%)
IVS	34 (55.7%)
**Prenatal change in size and number**	
No record of alteration	12 (/)
Progression	41 (83.7%)
No change	7 (14.3%)
Regression	1 (2.0%)
**Postnatal change in size and number**	
Lost at follow-up	5 (/)
Progression	0
No change	8 (36.4%)
Regression	14 (63.6%)

Severe hemodynamic changes occurred in 6.6% (4/61) of the fetuses. Six fetuses presented left/right ventricular outflow tract obstruction, and three of them progressed to pericardial and peritoneal effusion (Supplementary Figure 1C). Moderate tricuspid regurgitation with or without pulmonary hypertension was observed in two fetuses (Supplementary Figure 1D). In case 6, the gravida had been diagnosed as TSC clinically, she underwent sonographic screening ahead of schedule at 16 weeks of GA. The fetus appeared to have a ventricular hyper echo accompanied by severe congenital cardiac structural deformities, which were directly related to a poor perinatal outcome rather than a TSC-associated prognosis. All subjects did not detect arrhythmia throughout pregnancy. MRI was performed from 20 to 36 weeks of gestation in 53 fetuses, 25 of them found SENs and cortical tubers. Three cases showed lateral or bilateral ventriculomegaly. Detailed information is contained in Table 2.

**Table 2:** Characteristics of 61 cases of prenatally diagnosed cardiac rhabdomyoma (CR).

**No**.	**Age**	**GA**	**Number of CR**	**Location of CR**	**Max diameter**	**Change intrauterine**	**Complication**	**MRI**	**Origin**	**Mutation**	**ACMG**	**Perinatal outcome**
p1	32	34	Multiple	LA RA LV RV	8.2	Increase	TR	Positive	*De novo*	TSC2	Likely pathogenic	TOP
p2	31	22	Multiple	LV RV	9.1	/	No	Positive	Parental	TSC1	VUS	^#^Fetal reduction
p3	20	35	Single	IVS	Unknown	Increase	No	Negative	*De novo*	TSC1/2	VUS	Liveborn
p4	23	23	Multiple	LV IVS	17	Increase	No	Positive	*De novo*	TSC2	VUS	TOP
p5	27	26	Multiple	LV RV IVS	12	Increase	No	Positive	*De novo*	TSC2	Likely pathogenic	TOP
p6	34	16	Multiple	LV IVS	Unknown	/	^*^Mutiple cardiac structural abnormalities	/	maternal	TSC2	Likely pathogenic	TOP
p7	21	30	Multiple	RV	13.9	No change	No	Negative	*De novo*	TCS2	VUS	Liveborn
p8	36	26	Multiple	LV RV	16.6	Increase	No	Positive	*De novo*	TSC2	VUS	TOP
p9	24	28	Single	LV	14	Increase	No	Negative	No	negative	/	Liveborn
p10	27	24	Multiple	LV RV	9.4	Increase	Partial LVOTO	Negative	*De novo*	TSC2	Likely pathogenic	TOP
p11	25	22	Multiple	LV IVS	8.8	Increase	No	Positive	Parental	TSC2	Likely pathogenic	TOP
p12	26	30	Single	LV	22	No change	No	Negative	No	negative	/	Liveborn
p13	28	23	Single	LV	5.5	Regression	Permanent left superior vena cava	Negative	No	negative	/	Liveborn
p14	29	35	Multiple	LV RV IVS	10.2	Increase	No	Positive	*De novo*	TSC2	Likely pathogenic	TOP
p15	28	27	Single	LV(MV)	12	Increase	No	Negative	No	Negative	/	Liveborn
p16	26	30	Multiple	LV IVS	10.3	Increase	No	Positive	*De novo*	TSC2	pathogenic	TOP
p17	25	24	Single	IVS	10.3	Increase	Partial RVOTO	Negative	No	negative	/	Liveborn
p18	20	31	Multiple	LV RV IVS	23	No change	No	Negative	Parental	TSC2	VUS	Liveborn
p19	26	24	Single	IVS	9	/	No	Negative	Maternal	TSC2	VUS	Liveborn
p20	25	28	Multiple	LV IVS	15.5	Increase	No	/	Maternal	TSC2	Likely pathogenic	TOP
p21	19	34	Single	LV	32.2	No change	Partial LVOTO	Negative	No	negative	/	Liveborn
p22	28	24	Multiple	LV IVS	16.2	Increase	LVOTO	Negative	Parental	TSC1	VUS	TOP
p23	26	32	Multiple	RV IVS	14	Increase	No	Positive	Maternal	TSC2	Likely pathogenic	Liveborn
p24	33	24	Multiple	LV RV IVS	13.5	Increase	No	Positive	*De novo*	TSC2	Likely pathogenic	Liveborn
p25	29	26	Single	LV	28.1	/	Pericardial effusion;peritoneal effusion	Negative	No	Negative	/	Still birth
p26	33	23	Multiple	LV IVS	8	Increase	No	Negative	No	Negative	/	Liveborn
p27	26	26	Single	IVS	10.8	Increase	No	Positive	*De novo*	TSC2	Likely pathogenic	Liveborn
p28	25	24	Multiple	LV RV	14	Increase	Partial LVOTO	Positive	*De novo*	TSC2	Pathogenic	TOP
p29	32	22	Multiple	LV RV IVS	9	Increase	Renal pelvis separation	/	*De novo*	TSC2	Likely pathogenic	TOP
p30	32	23	Multiple	LV(MV) IVS	4.3	/	No	Positive	*De novo*	TSC2	Likely pathogenic	TOP
p31	28	28	Single	IVS	14.1	/	No	Positive	*De novo*	TSC2	Likely pathogenic	TOP
p32	21	25	Single	RV	12.9	Increase	No	Negative	No	Negative	/	Liveborn
p33	34	22	Multiple	LV RV IVS	9	Increase	No	/	*De novo*	TSC2	VUS	TOP
p34	32	23	Multiple	LV IVS	7.5	Increase	No	/	*De novo*	TSC2	Likely pathogenic	TOP
p35	20	25	Multiple	LV RV	8	Increase	No	Negative	No	Negative	/	Liveborn
p36	29	28	Multiple	LV RV IVS	12.7	Increase	No	Positive	No	Negative	/	TOP
p37	22	24	Multiple	LV RV IVS	11	Increase	No	Negative	*De novo*	TSC2	VUS	Liveborn
p38	22	35	Multiple	LV RV	21.4	No change	TR; PH; right ventriculomegaly	Positive	*De novo*	TSC2	Likely pathogenic	Liveborn
p39	31	28	Multiple	LV IVS	10.1	Increase	pericardial effusion	Negative	No	Negative	/	Liveborn
p40	28	22	Multiple	LV RV IVS	13.3	Increase	No	Positive	*De novo*	TSC2	VUS	Liveborn
p41	40	24	Single	RV	9	Increase	No	Positive	*De novo*	TSC2	Likely pathogenic	TOP
p42	30	23	Multiple	LV IVS	8.6	/	No	/	*De novo*	TSC2	Likely pathogenic	TOP
p43	24	25	Single	LV(MV)	14	Increase	No	Negative	No	Negative	/	Liveborn
p44	22	25	Multiple	LV IVS	8.8	/	No	Positive	*De novo*	TSC2	Likely pathogenic	TOP
p45	32	28	Multiple	LV RV IVS	7.1	No change	No	Positive	*De novo* & parental	TSC1/2	Likely pathogenic	TOP
p46	24	24	Multiple	LV	13.5	Increase	No	Positive	*De novo*	TSC2	pathogenic	TOP
p47	22	28	Multiple	LV RV IVS	8.2	/	No	Positive	Maternal	TSC1	Likely pathogenic	TOP
p48	28	24	Single	IVS	15.4	Increase	No	Negative	No	Negative	/	Liveborn
p49	25	26	Multiple	LV	15	Increase	No	/	*De novo*	TSC2	VUS	TOP
p50	36	25	Multiple	LV RV	9.5	Increase	No	Negative	Parental	TSC2	Likely pathogenic	Liveborn
p51	36	30	Single	RV	14	No change	No	Negative	No	Negative	/	Liveborn
p52	31	32	Multiple	RV	14.3	Increase	TR	Positive	*De novo*	TSC2	VUS	TOP
p53	32	28	Multiple	LV RV	11.1	Increase	No	Positive	*De novo*	TSC2	Likely pathogenic	TOP
p54	23	25	Multiple	RV	9	/	No	Negative	Parental	TSC2	VUS	TOP
p55	29	28	Single	LV	5	Increase	No	/	*De novo*	TSC2	Likely pathogenic	TOP
p56	32	24	Multiple	RV IVS	10	Increase	No	Negative	*De novo*	TSC1	Likely pathogenic	^#^Fetal reduction
p57	31	32	Multiple	LV IVS	18.9	Increase	No	Positive	*De novo*	TSC2	Likely pathogenic	Liveborn
p58	34	28	Single	IVS	10.1	Increase	No	Negative	Parental	TSC2	VUS	Liveborn
p59	30	26	Multiple	LV	11.7	/	No	Positive	No	Negative	/	TOP
p60	17	20	Multiple	LV	11	/	LVOTO, peritoneal effusion, ABS	Negative	Maternal	TSC1	pathogenic	TOP
p61	27	22	Multiple	LV RV IVS	12.3	Increase	right duplex kidney	Negative	Parental12%	TSC2	Likely pathogenic	Liveborn

* *Associated with single ventricle, single atrium, common atrioventricular valve, and permanent arterial trunk*.

# *Associated with twin pregnancy, one is affected and the other is normal*.

*LA, left atrium; RA, right atrium; LV, left ventricle; RV, right ventricle; IVS, interventricular septum; LVOTO, left ventricular outflow tract obstruction; RVITO, right ventricular inflow tract obstruction; TR, tricuspid regurgitation; PH, pulmonary hypertension; TOP, termination of pregnancy*.

### Detection of *TSC1* and *TSC2* Variants

**Figure 2:**
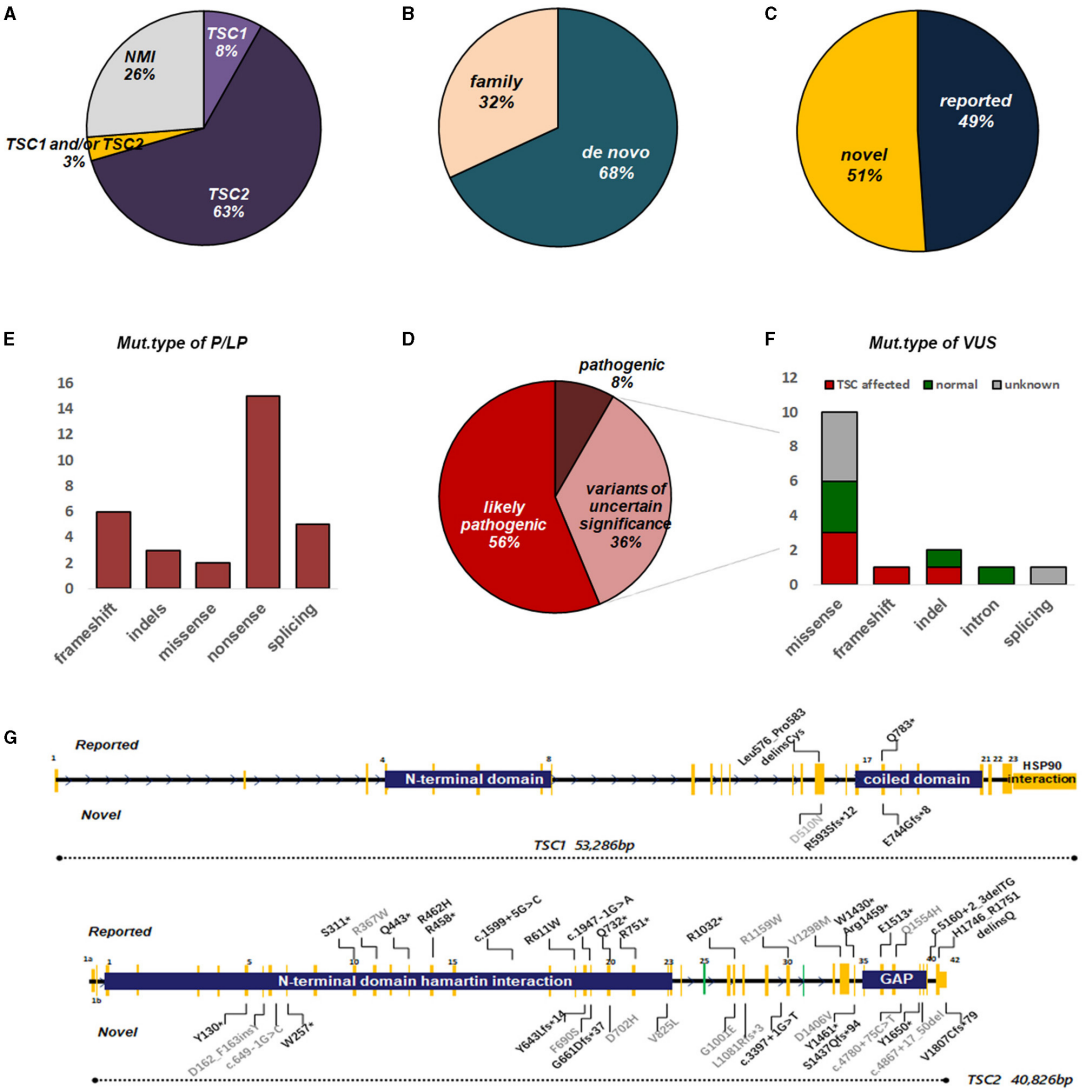
Overview of genetic testing results of TSC1/2 in fetuses with CR. **(A)** Percentages of TSC mutation found or no mutation identified (NMI) in fetuses with CR. **(B)** The proportion of the origin of TSC variation in fetuses with CR. **(C)** The ratio of the reported sites to the novel sites in TSC1 and TSC2 genes. **(D)** Among the identified mutations, pathogenic, likely pathogenic, or uncertain significance, TSC1/2 mutations are indicated. **(E)** Molecular consequences of mutations classified as pathogenic or possibly pathogenic. **(F)** Molecular consequences of mutations classified as VUS. Annotations include the following: **(G)** Distribution of mutation types in fetuses with CR(s). For TSC2, mutations were scattered throughout the gene with no enrichment in specific hotspots or domains, although there were several recurrent mutations, suggesting founder effects in certain populations. The number of mutations on the TSC1 gene was much less, mainly concentrated near the C-domain and interacting with tuberin. Annotations include the following: reported mutations (top); novel mutations (bottom); mutations of pathogenic/likely pathogenic (black); mutations of uncertain significance (gray). Splice site mutations are not shown here (see Supplementary Table 1). GAP, GTPase-activating protein.

In general, *TSC1* and *TSC2* variants were detected in 45 (Figure 2G) of 61 fetuses (73.8%), leaving 16 with no variant identified (NVI) (Figure 2A). Two-thirds of these variants were *de novo* (Figure 2B). Roughly 50% of these variants had not been reported previously (Figure 2C). Following ACMG Standards, we identified 30 variants of P/LP and 16 VUS (Figure 2D). The P/LP variant spectrum in *TSC1/TSC2* was 16 (34%) non-sense, 11 (23.4%) missense, 7 (14.9%) frameshift, 5 (10.6%) splice, 4 (8.5%) indels, and 2 (4.3%) introns (Figure 2E). When we focused on VUS, poor prognosis presented in all the truncating variant fetuses. Half of the missense/indel variants and all the intron variants tended to have a good outcome (Figure 2F).

### Genotype-Phenotype Correlation

The profile of genotype and phenotype in fetuses with CR(s) is depicted in Table 3. For the 30 cases identified with variations of P/LP: 90% (27/30) had multiple CRs. Overall, 80% (20/25) were cerebral MRI positive before birth, and TSC-associated signs appeared in 85.7% (6/7) of the live-born infants. Two babies with multiple CRs but normal brain MRI were born, one of which developed epilepsy at 6 months, and SENs appeared. In the fetuses carrying VUS: the incidence rate of multiple tumors was as high as 73.3% (11/15), yet cerebral lesions observed prenatally were much lower (46.7%). In total, 85.7% (6/7) of VUS without nervous findings had the normal phenotype as far as we followed up. Postnatal brain damage and neurological symptoms onset occurred in only one infant without MRI findings *in utero*. As to the group with NVI, multiple CRs were found in 31.2% (5/16), and 87.5% (14/16) had normal nervous imaging. In two NVI fetuses (p36, p59), cortical nodules proved by MRI supported the diagnosis of TSC clinically. Both of them underwent TOP, and the heart tissues testing confirmed the NVI result (data are not shown). In terms of the mutation genes, *TSC2* occupied a dominant position. Considering the *TSC2* pathogenic variants are related to a more severe phenotype than *TSC1* (9), we compared them in fetuses with CR(s). Both the frequency of TSC-causing mutations and number of CRs had no significant difference (*P* > 0.05). However, *TSC2* variants were more related to positive neuroimaging findings than *TSC1* (*P* < 0.05) (Table 4).

**Table 3:** The profile of genotype and phenotype in fetuses with CR(s).

**Genetic**	**Echocardiographic findings**		**MRI findings**	
	**Multiple CRs**	**Solitary CR**	**Positive**	**Negative**
P/LP	27	3	20	5
VUS	11	4	5	7
N	5	11	2	14
*P*	<0.05		<0.05	

**Table 4:** Phenotype differences between TSC1 and TSC2 in fetuses with CR(s).

	**Genetic classification**		**Echocardiographic findings**		**MRI findings**	
	**P/LP**	**VUS**	**Multiple CRs**	**Solitary CR**	**Positive**	**Negative**
*TSC1*	5	2	6	1	3	4
*TSC2*	26	15	34	7	22	10
*P*	>0.05		>0.05		<0.05	

### Clinical Outcome

Finally, 34 families decided to terminate the pregnancy. Among these, case 25 experienced intrauterine fetal death at GA of 26 weeks for severe hemodynamic alteration. A considerable tumor (28 × 28 mm) obstructed the left ventricular outflow tract and was complicated with pericardial effusion/peritoneal effusion. In the remaining 27 cases, postnatal data were available for 23 live births; four were lost at follow-up (Table 5).

**Table 5:** Prognosis information for live-born fetuses or those lost at follow-up.

**No**.	**Sex**	**Number**	**MRI**	**Variation**	**ACMG classification**	**TSC-associated lesions**	**CR change after birth**
p3	F	S	Negative	TSC1/2	VUS	No	4 M no change
p7	M	M	Negative	TCS2	VUS	No	7 M decrease
p12	F	S	Negative	Negative	N	No	4 M decrease
p13	M	S	Negative	Negative	N	No	3 M no change
p17	M	S	Negative	Negative	N	No	12 M regression
p18	F	M	Negative	TSC2	VUS	No	17 M decrease
p19	F	S	Negative	TSC2	VUS	No	12 M regression
p21	F	S	Negative	Negative	N	No	24 M decrease
p23	M	M	Positive	TSC2	LP	Epilepsy; multiple retinal hamartomas; hypomelanotic macules	2 M no change
p24	M	M	Positive	TSC2	LP	Epilepsy	3 M no change
p27	F	S	Positive	TSC2	LP	Epilepsy	2 M decrease
p32	F	S	Negative	Negative	N	No	8 M decrease
p35	M	M	Negative	Negative	N	No	2 M no change
p37	M	M	Negative	TSC2	VUS	Epilepsy (detected SENs afterbirth); hypomelanotic macules	8 M regression
p38	F	M	Positive	TSC2	LP	Epilepsy	2 M no change
p39	M	M	Negative	Negative	N	No	3 M decrease
p40	F	M	Positive	TSC2	VUS	Epilepsy	4 M no change
p43	M	S	Negative	Negative	N	No	15 M regression
p50	M	M	Negative	TSC2	LP	No	18 M regression
p51	M	S	Negative	Negative	N	No	4 M no change
p57	M	M	Positive	TSC2	LP	Epilepsy	4 M decrease
p58	M	S	Negative	TSC2	VUS	No	4 M decrease
p61	M	M	Negative	TSC2	LP	Epilepsy (detected SENs afterbirth); moderate intellectual disability; hypomelanotic macules	12 M decrease
**Lost at follow-up**							
p9	/	S	Negative	Negative	N	/	/
p15	/	S	Negative	Negative	N	/	/
p26	/	M	Negative	Negative	N	/	/
p48	/	S	Negative	Negative	N	/	/

**Figure 3:**
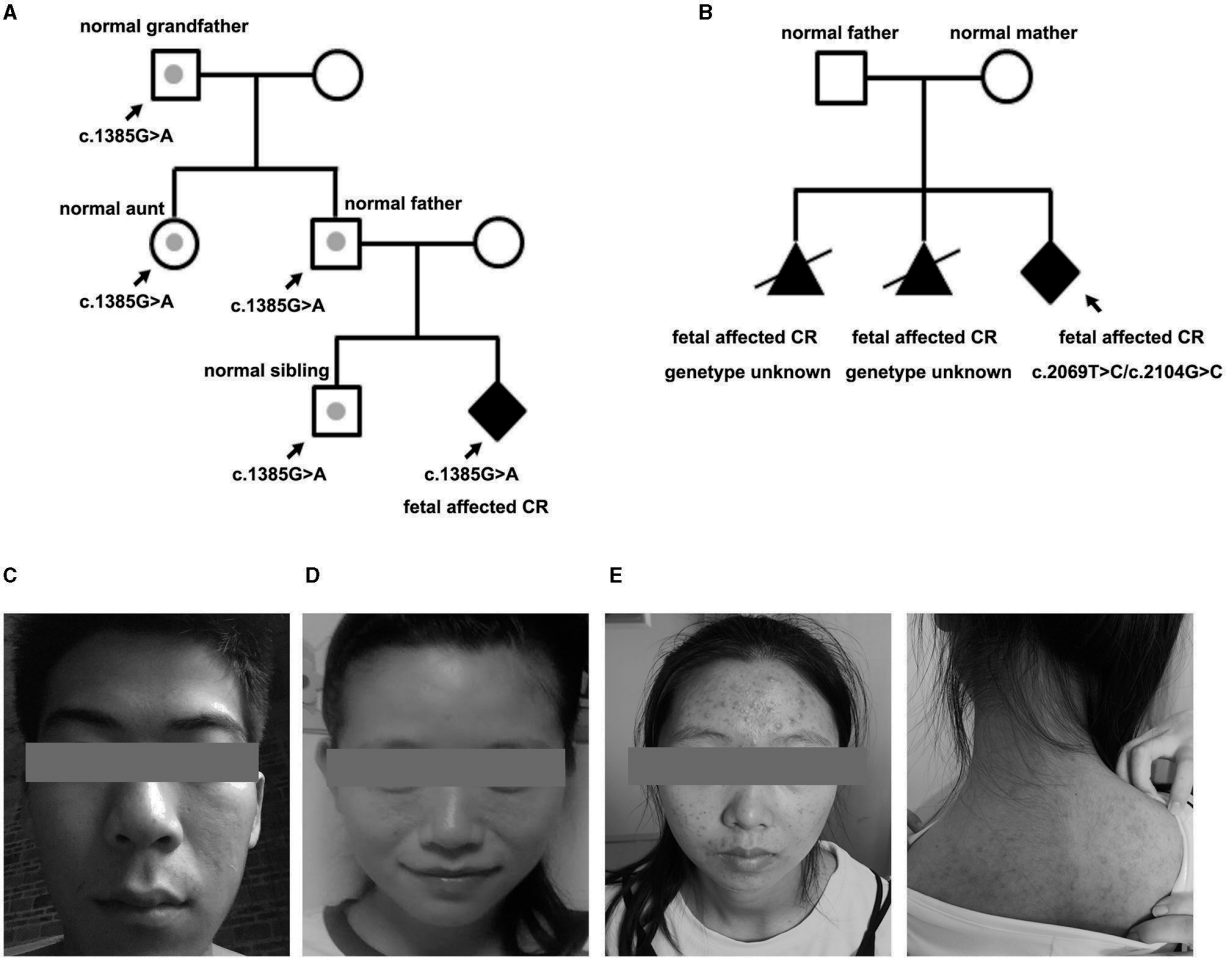
Pedigree of the family variation in TSC2 in a particular family and photographs. **(A)** The CR-affected fetus had a heterozygote LP variant, which had also been confirmed in the normal relatives (case 50). Gray dots indicate carrying the LP variant but without a TSC phenotype. **(B)** Pedigree of case 33 is shown with potential gonadal mosaicism. The black triangle indicates the aborted fetuses suffering from CR but without a genetic test. **(C)** Father of fetus 61 who was identified with 12% mosaicism of the TSC-causing mutation, only having subtle signs of facial angiofibroma. **(D)** Mother of case 47 who carried a TSC1 mutation but only showed subtle signs of facial lesions. **(E)** Face and back of the mother of case 20. The facial angiofibroma was occasionally accompanied by infection, but she thought it was intractable acne and never received standardized diagnosis and treatment.

Among the 23 newborns (15 boys, eight girls), the tumor decreased or regressed in 15 of them (10 boys, five girls) no <3 months after birth. Overall, nine of the 23 newborns were negative for *TSC1/2* mutation, seven for VUS, and seven for LP. All the brain MRI positive fetuses presented neurological symptoms within 1 year after birth. MRI imaging was standard in p37 and p61 before delivery, for those who carried a VUS and an LP mutation, respectively. While new SENs were found in both when epilepsy occurred at about half a year old. All the fetuses with LP variants were finally proved to have brain lesions except one (case 50). Case 50 showed a known *TSC2* missense variant (c.1385G>A; p.R462H), which had already been classified as a likely pathogenic marker for TSC (clinvar). NGS-trio analysis of the family confirmed the father also carried this mutation without the TSC-associated phenotype. What is more, we also detected c.1385G>A in a healthy sibling, aunt, and grandfather (Figure 3A).

For case 33, it was the third time they had fallen pregnant with a fetus with CR. NGS-trio identified two *de novo* missense *TSC2* mutations in the fetus (Figure 3B). The couple decided to TOP immediately without further evaluation of the neurological examination. We had no more information to clarify the pathogenicity of the two mutants. Considering the uncertain significance of these variants and the possibility of gonadal mosaicism, risk of recurrence is still high.

### Work-Flow Suggestion

**Figure 4:**
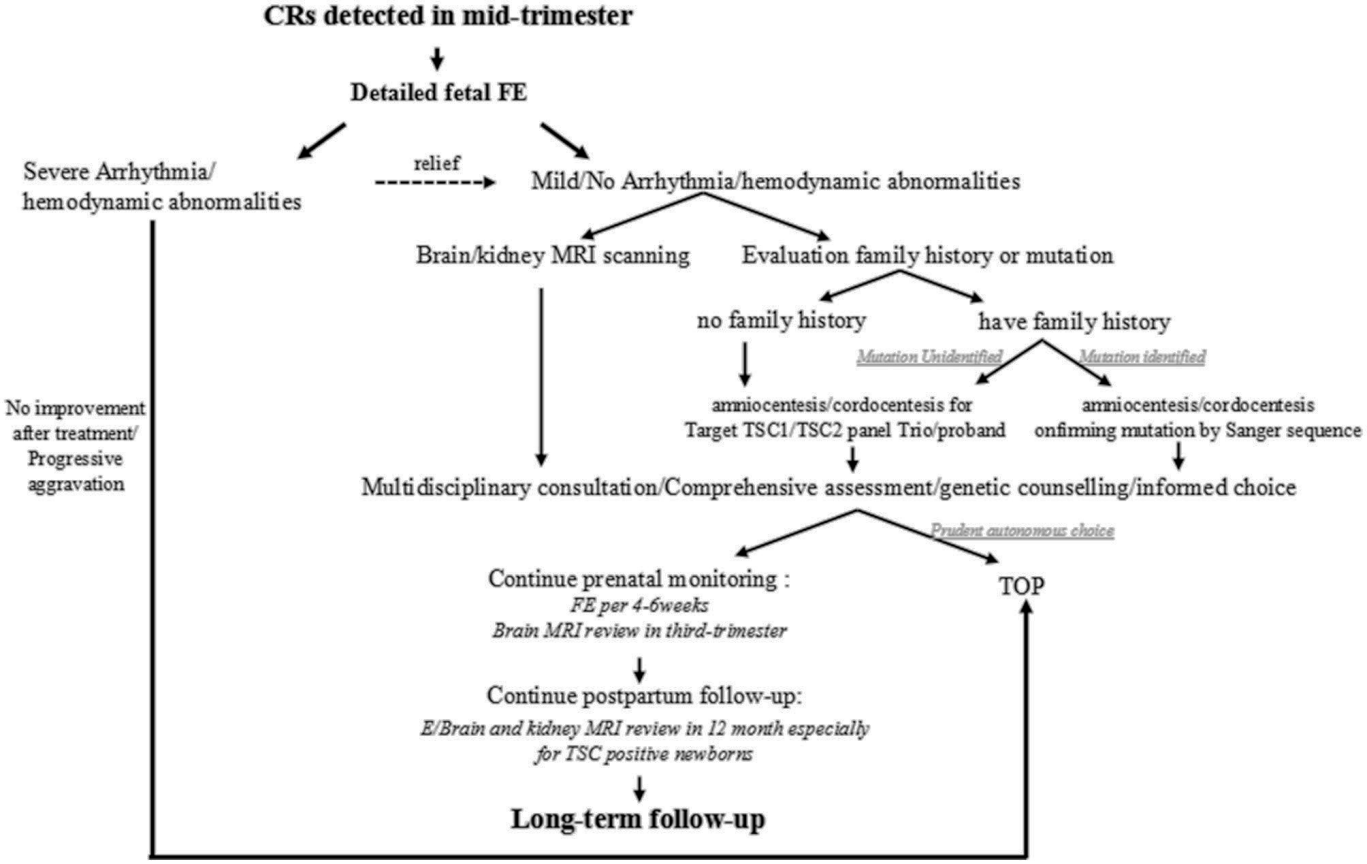
Proposed clinical workflow following CR(s) for other clues to TSC.

Thinking over the fact that most intrauterine CRs were first observed coincidentally through a level III ultrasound during 22-26 GA except for the one who had detailed family history, we proposed a simplified clinical work-flow for this situation based on our experience (Figure 4). When cardiac tumors are detected in the mid-trimester, a detailed fetal echocardiography examination is recommended firstly to assess the cardiac structure and function and to give proper treatment. Then, an MRI for the nervous system should be performed as well as family history collection. For CRs in fetuses whose family history is ambiguous or mutation unknown, targeted NGS-Trios could be the most effective and time-saving path when searching the molecular etiology. Genetic counseling should be provided after a comprehensive analysis of clinical symptoms, imaging, and molecular results, which are essential for pregnancy decision or clinical management. Follow-up before and after birth are both indispensable. Benefiting from the multidisciplinary approach, 68.9% of fetuses (42/61) with CRs could receive a precise clinical/genetic diagnosis, or a known outcome, which turned out to be potentially clinically actionable.

## Discussion

When cardiac rhabdomyomas are detected in a fetus, both the perinatal outcome dominated by tumor space occupation and the long-term prognosis related to TSC are critical, but tricky. We tried to assess perinatal risk and prenatal diagnosis of TSC with CR as the initial symptom through a multidisciplinary approach, which opens up the possibility of relatively reliable assessment for numerous *TSC* VUS.

### The Influence of Tumor on Perinatal Outcome

Outflow tract obstruction was not rare as it occurred in ~10% of this study. But severe hemodynamics disorders were scattered partly because multiple CRs, which accounted for 70% in this study, tended to be smaller (10) and chamber-located (11). Although 83.7% of tumors increased by the stimulation of maternal estrogen prenatally (12), the increment reduced after the 32nd week (13). So the masses were at their most significant in the mid/last trimester and therefore required more rigorous and careful monitoring. The earliest ultrasonic observable time of CR was reported at 15 weeks of GA (14), but the exact time of tumorigenesis is difficult to deduce. Case 6 provided some clues for early events in tumor formation. We conjectured that early-onset CRs might be manifested as the diffuse ventricular hyperechoic type and could affect the normal remodeling process in the heart. Thus, the diagnosis of TSC in some complex cardiac structural malformation fetuses might be underestimated or ignored. Although the proliferation of CRs seems self-limiting and independent of TSC gene alteration, tumorigenesis might result in gene instability, such as a loss of heterozygosity or a second variant (15). Supporting evidence from targeted-deep sequencing in TSC-associated hamartoma samples found that roughly 2/3 of hamartomas from TSC individuals harbored two TSC hits (16). We tested CR tissues in two aborted TSC fetuses, and both the mutations were consistent with their amniotic fluid results (data are not shown), suggesting a germline origin of mutations. Therefore, strengthening echocardiography monitoring and reassessing tumors in middle pregnancy is indispensable.

Some postpartum studies revealed that up to 23% of children with CRs may present with arrhythmia (17), which may be related to abnormal conduction of electrical excitement in tumor tissues near the atrioventricular junction (18). Tetsuya et al. reviewed 20 fetuses with CRs and speculated that, regardless of number and location, tumor diameter >30 mm is associated with postnatal arrhythmia (19). We observed no arrhythmia in all the subjects including the one who suffered a single huge tumor located in the LV which exceeded 30 mm.

### The Influence of Tumor-Related TSC on Long-Term Prognosis

The influence of CR-related TSC is a decisive factor for long-term prognosis, although this is contested. NGS facilitates the detection process but leaves the problem of variant recognition. We amplified the detection yield to 73.8% (including mutations of P, LP, and VUS), which was much higher than 10 years before (20). Chen et al. (11) identified *TSC1/TSC2* P/LP variants in 69.8% of fetuses with CR which seemed more instructive. We speculate that there was a selection bias for the inclusion criteria. Rhabdomyomas could not be fully confirmed pathologically until fetus tissues were obtained. Thence the specificity of ultrasound for the diagnosis of CR is the main reason for the difference in the detection rate between different cohorts. Some other benign cardiac tumors, such as teratomas, fibroids, hemangiomas, or hamartomas, might pull down the variants detection rate. In order to avoid this selection bias as much as possible, we differentiated these tumors by rigorous fetal echocardiogram selection according to various characteristics before inclusion (21). The majority of teratomas arise from the pericardium and are of mixed echogenicity with cystic or calcified structures. Almost all are associated with pericardial effusion. Fibroid and hemangioma were often higher in size and grew fast, with or without calcification. Myxomas may attach to the atria and swing with the rhythm; also, malignant tumors are rare (2). Słowińska et al. confirmed the feasibility of a similar algorithm in early diagnosis, before seizure onset, of CR children (22). The cohort composed of newborns or infants within the first 16 weeks of age, so the algorithm was mostly based on postpartum clinical signs, supplemented by brain MRI, echocardiography, skin examination, and genetic testing. Compared with our study, whose objects were all fetuses, antenatally observable phenotypes were extremely limited. Therefore, we weighted the results from genetic assessments in prenatal diagnosis. The accuracy of the algorithm for VUS is vital information that affects family decision-making.

*Family phenotype collection* is in the top priority of genetic counseling. About 32% of mutations identified in this cohort were inherited from parents without a typical phenotype. Clinical expression is hugely variable. Some subtle signs, mostly skin manifestations, were easily overlooked or misdiagnosed, and 3–6 outpatient visits were needed to obtain a preliminary diagnosis in China. p20 and p23 had both suffered from “acne” for decades, which were actually atypical facial angiofibromas for TSC (Figures 3D,E). Mosaicism is another essential factor affecting phenotype, especially of mild cases with NVI by conventional testing, which afforded 7.5% of patients with a clinical TSC diagnosis (23). Variant allele fraction (VAF) in the blood was positively correlated with the number of major features (23, 24). Interestingly, low-level mosaicism (0–10%, median 1.7%), which likely arises from a later postzygotic variant, had a milder and distinct clinical phenotype in comparison with other TSC series, with similar facial angiofibromas (92%) and kidney angiomyolipomas (83%) (25). VAF of the p61 husband was 12%, he showed an unnoticeable asymmetric facial angiofibroma (Figure 3C). However, the p61 infant, who carried the heterozygous variant, not only had facial lesions but also had intellectual disability, and epilepsy presented at 6 months. The difference in performance is not only closely related to the mosaic level, but may also be affected by factors such as the structure of the variant, the tissue distribution, and the penetrance rate. Cutaneous signs are the most common signs in TSC phenotype series, and vice versa, the variant of *TSC1/TSC2* was the most frequently detected in skin lesions (26). Thus, dermatological consultations or even skin biopsy sequencing are necessary for asymptomatic parents whose variants are accidentally discovered by NGS-trio to confirm the diagnosis, especially when encountering a VUS.

Penetrance is high but not absolute, which also influences the assessment of pathogenicity of variants. The *TSC2* p.R462H substitution has been annotated as likely pathogenic by LOVD. *In vitro* experimental studies showed that the missense mutation had an effect on TORC1 activity and interfered with the formation of the *TSC1-TSC2* complex, and resulted in an unstable protein and impaired protein function (27). The mutant was not recorded in population databases (rs45494392, ExAC no frequency) but uniquely presented in the p50 pedigree (Figure 3A). The fetus was negative for neuroimaging findings during the whole pregnancy. All indexes point to benign prognosis, which had been confirmed in follow-up. Integrated genetic information in trios, and imaging results may enable early implementation of further diagnostic investigations, perinatal surveillance, and family counseling, especially for couples with no signs of TSC phenotype on clinical examination.

For post-test analysis in the NGS era, it was common for phenotypic fetuses to show VUS of *TSC1/2* despite over 10,000 recorded variants. Some other angles of view need to be introduced to aid further evaluation. Pathological changes in the CNS are observed in almost all TSC individuals, and ~80–90% of them present with cortical tubers and/or SENs, and seems equally familiar in *TSC1* and *TSC2* pathogenic mutations (26). However, antenatal neurological manifestations are easily overlooked and mostly checked after CR detection during the second-trimester scanning when the pathological changes in the CNS might have gone a long way. Saada et al. (14) indicated that the characteristic cerebral lesions of TSC may form as early as 10–20 weeks of embryonic development. As recently as 2007, Mühler et al. preliminarily explored the application of fetal cerebral MRI in sonographically proven CR (28). Here, we disclosed that neuroimaging finding was a much more specific indication of genetic TSC and was proportional to poor prognosis. *TSC2* mutations seemed more likely to have neuropathy than *TSC1* in prenatal fetuses. In combination with fetal MRI, neurological lesions were found in two NVI cases and five *TSC2* VUS cases, for whom a clinical diagnosis of TSC was made. Exceptionally, in one case with VUS, neurological symptoms occurred at 16 weeks after birth. Benefiting from prenatal MRI scanning, some VUS, or even NVI turned out to be potentially clinically actionable. Neuro MRI should be included in the clinical diagnosis process to achieve regular monitoring in a fetus with CR.

Besides, the possibility of parental gonadal mosaicism cannot be overlooked in a typical CR fetus with more than one proband sibling. The reported incidence of germ-line mosaicism ranges from 2 to 5% (29). Case 33 here was a family with two fetuses with CR(s). For this pregnancy, two missense variations of *TSC2* were detected in the fetus but absent in both parents. Although both of the mutations were pathogenically ambiguous, it was strongly suspected to be germ-line mosaicism in the couple. It implied that the recurrence risk of unaffected parents who have had an affected child would increase because of germ-line mosaicism, and families with high suspicion of this should be advised to undergo prenatal diagnosis for each pregnancy.

## Conclusion

We recommended strengthening fetal heart examination during middle and late pregnancy, especially for pregnancy with a family history of TSC. Fetal echocardiography is the preferred method for detecting fetuses with CR. Neuroimaging findings are a more sensitive indicator compared to the number of CR to imply the pathogenicity of *TSC1/2* variants and a poor prognosis. Combined with neuro MRI and genetic testing, it facilitates early detection of TSC and is of great significance for perinatal management and prognostic guidance. The multidisciplinary approach could allow nearly 70% of fetuses with CR(s) to be potentially clinically actionable.

## Limitation

Our study still has some limitations that warrant further consideration. First, the pathological information of these cardiac tumors is insufficient and lacks autopsy information. Second, a limited number of cases identified *TSC1* mutations, making it difficult to assess the relationship between CR, genotype, and neuroimaging changes for this subpopulation of TSC. Third, the clinical manifestations of TSC can be mimicked by mutations in many genes. Sequence coverage was restricted to exons and adjacent intronic mutations, which perhaps missed mutations deep within introns or in other unknown regulatory regions.

## Data Availability Statement

The data presented in the study are deposited in the NCBI repository (https://www.ncbi.nlm.nih.gov/sra/PRJNA742183), accession number (PRJNA742183).

## Ethics Statement

The studies involving human participants were reviewed and approved by the Ethics Committee of the Guangdong Women and Children Hospital. The patients/participants provided their written informed consent to participate in this study. Written informed consent was obtained from the individual(s) for the publication of any potentially identifiable images or data included in this article.

## Author Contributions

YQ carried out all the data analyses, participated in the design of the work, and wrote the draft. HD and YZh collected all clinical data. YH, YZe, LY, and LL participated in molecular genetic test work. AY designed the work and revised the manuscript. All authors have materially participated in the study and manuscript preparation, critically reviewed the manuscript and provided final approval for submission, and agreed to be accountable for all aspects of the work, ensuring the accuracy and integrity of the publication.

## Conflict of Interest

The authors declare that the research was conducted in the absence of any commercial or financial relationships that could be construed as a potential conflict of interest.

## Publisher's Note

All claims expressed in this article are solely those of the authors and do not necessarily represent those of their affiliated organizations, or those of the publisher, the editors and the reviewers. Any product that may be evaluated in this article, or claim that may be made by its manufacturer, is not guaranteed or endorsed by the publisher.

## Supplementary Material

Supplementary Figure 1 Fetal echocardiography. **(A)** Four-chamber view of p21 at 19 weeks' gestation, a single giant rhabdomyoma (32.2 × 22.5 mm) presented on the left ventricular, with clear border, uniform internal echo, and no obvious relationship with the posterior leaflet of the mitral valve. **(B)** Multiple rhabdomyomas scattered in LV, RV, and IVS at 32 weeks' gestation of p29, the diameter ranged from 2.2 to 9.0 mm. **(C)** In the four-chamber view, a large rhabdomyoma was visible in LV, and a small amount of fluid can be seen in the pericardial cavity, about 6.0 mm deep. The p25 fetus was concurrent with peritoneal effusion and stillbirth at 26 weeks' gestation. **(D)** Four-chamber view of p38 at 35 weeks' gestation, the tricuspid valve is well-open, while moderate regurgitation can be detected when closed.

Supplementary Table 1 Genotypic and phenotypic features of patients with germline mutation of TSC1/2.

## Abbreviations

CR: cardiac rhabdomyoma
TSC: tuberous sclerosis complex
MRI: magnetic resonance imaging
VUS: variations of uncertain significance
P: pathogenic
LP: likely pathogenic
SENs: subependymal nodules
CNVs: copy number variations
TOP: termination of pregnancy
NVI: no variant identified
NGS: next generation sequence.
