# Supplementary figures and images for: A Multidisciplinary Approach in Prenatal Diagnosis of TSC With Cardiac Rhabdomyoma as the Initial Symptom
Source: Front Pediatr. 2021 Aug 27;9:628238. doi: 10.3389/fped.2021.628238 (PMC8429840; doi:10.3389/fped.2021.628238)

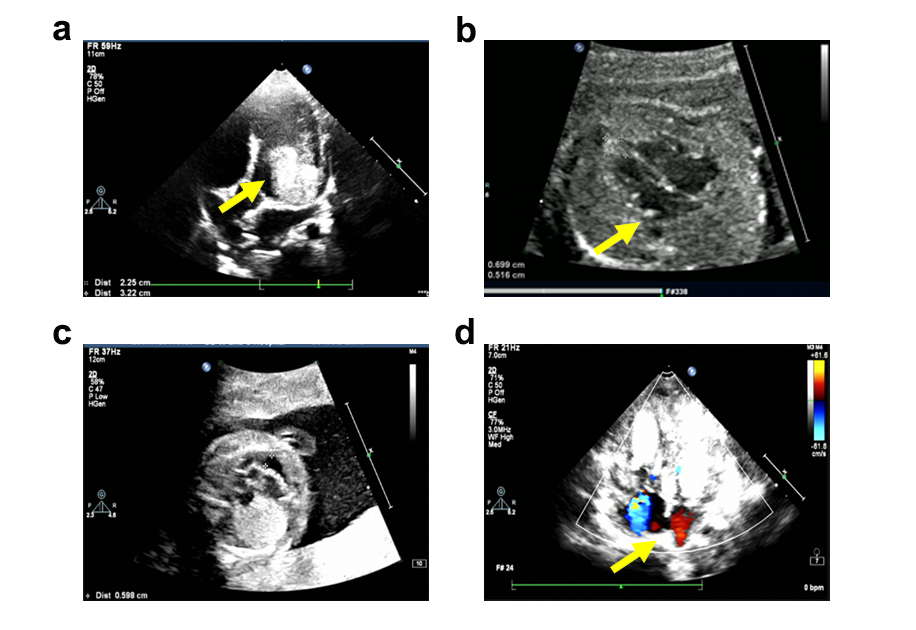

Supplement: Supplementary Figure 1 — Fetal echocardiography. (A) Four-chamber view of p21 at 19 weeks' gestation, a single giant rhabdomyoma (32.2 × 22.5 mm) presented on the left ventricular, with clear border, uniform internal echo, and no obvious relationship with the posterior leaflet of the mitral valve. (B) Multiple rhabdomyomas scattered in LV, RV, and IVS at 32 weeks' gestation of p29, the diameter ranged from 2.2 to 9.0 mm. (C) In the four-chamber view, a large rhabdomyoma was visible in LV, and a small amount of fluid can be seen in the pericardial cavity, about 6.0 mm deep. The p25 fetus was concurrent with peritoneal effusion and stillbirth at 26 weeks' gestation. (D) Four-chamber view of p38 at 35 weeks' gestation, the tricuspid valve is well-open, while moderate regurgitation can be detected when closed. [file Image_1.tif]
